# Supplementary material for: “A good day is just being able to breathe”: Aligning COPD research with patient needs, a qualitative study
Source: PLoS One. 2025 Sep 16;20(9):e0331403. doi: 10.1371/journal.pone.0331403 (PMC12440184; doi:10.1371/journal.pone.0331403)
Supplement: S1 File — The final interview guide was used with participants. (PDF) [file pone.0331403.s001.pdf]

Supplemental File 2: Interview Guide

Introduction/Verbal Consent:

Hello, my name is ________, and I am a part of the research team at [lab conducting the study]. We are conducting Zoom interviews with patients living with COPD and their caregivers. Your participation in this interview is completely voluntary, and you can choose not to participate or answer any questions that I may ask you. The information we discuss will be confidential and not a part of your medical record. We will use the information we gather to understand how to better support future participants when they join a clinical research study about COPD. Are you still interested in participating? The interview should take no more than 45 - 60 minutes. Okay, let's begin.

Welcome

- Thank you again for coming and taking the time to participate in this project
- The purpose of this project is to explore your experiences and preferences about research that studies how we treat and manage COPD. We are interested in your reflections on the different components of the studies and how they made you or might make you feel or how they did or could impact your life.
- This information is being collected with the goal of improving how we conduct research studies and care for patients like you in our community.
- We want to make sure we understand all of the things that are important to a person living with COPD when thinking about research studies. We'd like to learn more about what you care about as a patient/caregiver of a patient living with COPD and what you think is less important.
- This is a qualitative study where the structured interviews will be analyzed for common themes and ideas between different participants. There are no right or wrong answers.

Ground Rules

- This interview is purely for research purposes. It will have no impact on your health care.
- We are recording the conversation so that we can transcribe it later
- There is no silly question or response, and everything said in this room is confidential
- When we publish this project, no identifying information will be published regarding any individual person
- You may choose not to answer any question at any time, and you may leave at any time.
- Your participation is entirely voluntary.

Interview Questions:

1. Overall Experience- *Patients Only*

2. When you think about your COPD, what day-to-day things are most important to you?
    - *Prompt: Is there a certain activity or task that helps you know how your COPD is doing that day?*
        1. *Prompt: ease of getting up stairs or completing a routine task?*
        2. *What does a good day living with COPD look like to you?*
3. What does a good week living with COPD look like to you?
4. What does a good month living with COPD look like to you?
5. What does a good year living with COPD look like to you?
    - *Prompt: If you had to focus on one activity to maintain, what would that be?*
    - *Prompt: How can you tell that you're doing "really well"?*

6. Overall Experience- *Caregivers Only*
    a. In your role as a caregiver, what kind of burdens did you experience while trying to manage your X's disease?
    b. What types were hardest for you?
    c. Were there times/years in her disease that were easier or harder for you? (ebb and flow depending on exacerbations, harder as the disease progressed, easier because more comfortable)
7. Study Participation (Previous study participants only)
    a. How are you doing since you participated in the study?
    b. How was your overall experience with the study?
    c. Did the study have an impact on your day-to-day life? If so, how?
    - *Prompt: What aspects did you find most helpful or challenging?*
    - *Prompt: Were there any specific changes in your daily routine?*

    d. After the study, did you feel more confident about managing your COPD? Did the study help you with this?
        - *Prompt: Can you give examples of how your health management changed?*

    e. Based on your experience with the study, what do you think we should focus on in future research to better help patients?
    f. How did you first hear about the study?
        - *Prompt: what made you first interested in participating in the study?*
        - *Prompt: Was it through a healthcare provider, friend, or another source*

    g. What did you think of the instructions while you participated in the study?
        - *Prompt: Were there any specific instructions you found confusing or helpful?*

    h. What did you think about the recruitment process?
        - *Prompt: Were there any things that made you feel comfortable or uncomfortable? Confusing or straightforward? How did signing up for the study go? Easy? Difficult?*
        - *Prompt: if you were to design the study for people like yourself, how would you reach out to people? How would do outreach to your friends/people you know with COPD?*
        - *Prompt: Is there another way you would prefer to learn about research studies*

- *e.g. through your physician or in the patient portal?*

i. Did your study have an app?

- *Prompt: If yes: Did you find it (the study app) helpful? Were you able to navigate the app easily?*
- *Prompt: What features did you find useful or challenging?*
- *Prompt: Did you encounter any difficulties while using the app? If so, can you describe where you felt stuck?*

j. What did you think of the language of any surveys or instruments?

- *Prompt: Were they too long? Was the language used easy to understand?*
- *Prompt: What aspects of the surveys were particularly challenging?*

k. Did the study team provide support if you needed help with the study?

8. Virtual versus in-person participation (Facilitator should prompt: sometimes interventions are offered in person, like in a hospital or clinical, or at home, through a video or app on your phone. One example of this in COPD is pulmonary rehabilitation.

a. Have you used a virtual program? *(if not, can skip remained of section)*

b. How was your overall experience with the program?

- Did you have any hesitations or excitement about participating?

c. Before starting the digital or virtual, what were your expectations or concerns about that type of program?

- *Prompt: What were the most valuable aspects of this program for you? How do you feel after using the program? Does anything specific come to mind?*

d. Did you face any challenges during the digital program?

- *Prompt: Were there moments when you felt uncomfortable?*
- *Prompt: were there components of the technology that made it difficult for you to participate in the activities*

e. How did you feel about your program being digital?

- *Prompt: would you have preferred that it be in person*
- *Prompt: What are the pros and cons of digital versus in-person services like telepulmonary rehabilitation?*

9. Participation and Withdrawal

a. What might make you withdraw from a research study? Why?

b. Was there ever a time you felt like withdrawing from the study?

a. Prompt: Did you find it difficult to manage alongside your other commitments?

10. Offboarding and Future Participation

a. If you have participated in a clinical research study, how was the offboarding process (stopping the study) experience for you?

- *Prompt: Did you feel prepared to transition out of the study?*
- *Prompt: If you were to conduct a study like this, how would you let participants know their participation in the study was coming to an end?*

b. Would you encourage your family or friends to participate in future studies?

- *Prompt: What factors influence your decision to recommend or not recommend participation?*

11. When thinking about studies you have participated in, in your opinion, where do you think we need to improve, and what do you think we are doing well?
    a. *Prompt: Can you provide specific examples for both?*

12. One of our goals is to have study participants more actively involved in not just doing the study activities (like wearing a Fitbit or answering surveys), but also designing the study. Participants might be involved in designing an intervention or what is measured to decide if the intervention is "helpful."
    b. What do you think is the best way to involve participants in studies in this way?
        i. *Prompt: examples might be doing interviews like this or presenting at places where a lot of eligible participants might be like at support groups or clinics.*
    c. Would you be interested in being a "community consultant" for future research studies? The role of community consultants is to give feedback about a study's approach and design, much like you're doing now, but typically before the study starts. You do not need to commit to this now.

13. If you were asked to participate in a study where we would be collecting information that might have implications on your health but will not be monitoring it in real-time what would you think about that?
    - *Prompt:* For example, if we were running a study where we used Fitbit data to see if you are starting to get sick. Because we are still learning, we did not alert you or your doctor because it might have been a false alarm. How would you feel about that

14. Who else do you think we should talk to about what is important about COPD research? Do you have any go-to personalities that you listen to? Do you have any support groups?
Prompt: a supportive organization? Someone from the hospital who is involved in your care? People who have family members living with COPD?
