# Supplementary material for: “A good day is just being able to breathe”: Aligning COPD research with patient needs, a qualitative study
Source: PLoS One. 2025 Sep 16;20(9):e0331403. doi: 10.1371/journal.pone.0331403 (PMC12440184; doi:10.1371/journal.pone.0331403)
Supplement: S1 Fig — Profiles of each participant were created to represent their perspective towards COPD-focused research, including key barriers and facilitators to engagement. (PDF) [file pone.0331403.s002.pdf]

Supplemental File 1

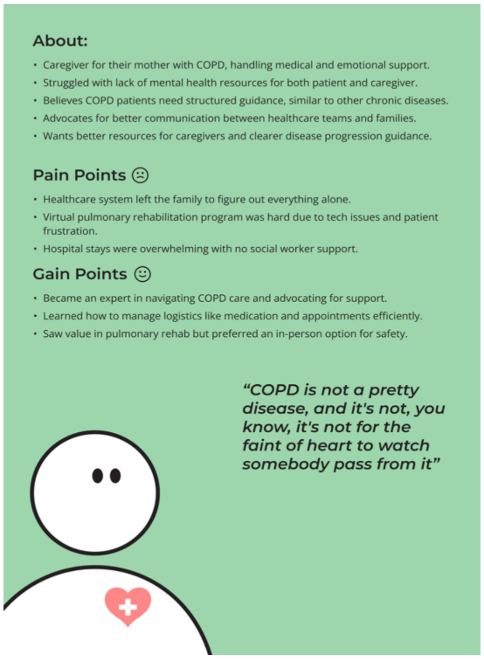

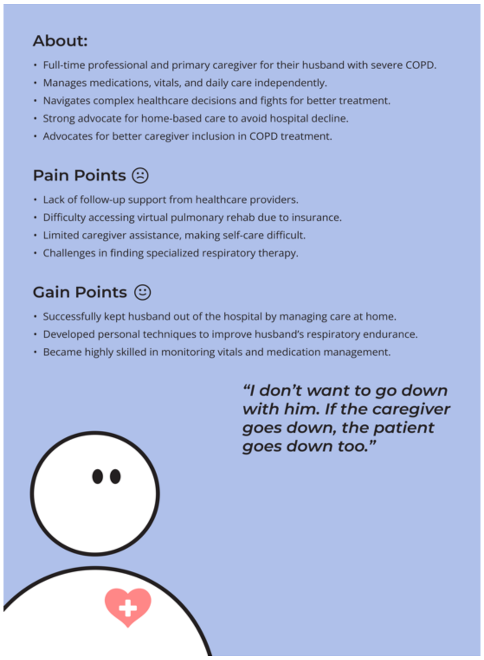

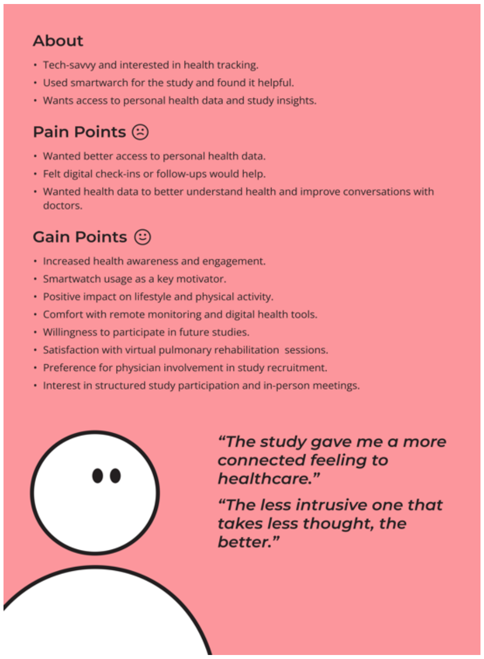

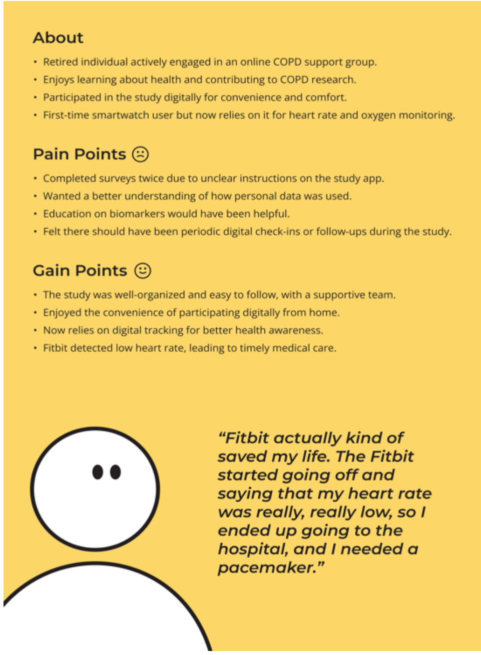

About

- Actively participates in research, eager to share experiences to improve patient care.
- Comfortable using technology.
- Takes initiative in managing health, exercising despite difficulties.
- Prefers direct communication like calling.

Pain Points ☹

- Experienced a COPD exacerbation soon after the study ended, leading to multiple rounds of prednisone and antibiotics.
- Found it difficult to continue regular exercise without structured support from the virtual pulmonary rehabilitation program
- The transition out of the study felt sudden, with little time to prepare or adjust.
- Post-study support group lacked medical professionals, reducing access to expert advice.

Gain Points ☺

- Regular check-ins and education helped with COPD management and provided emotional support.
- Formed a patient-led support group that continues to provide emotional and practical support.
- Learned effective breathing techniques, improving breathing control and daily activities.
- Smart health devices helped monitor oxygen levels and exercise progress.

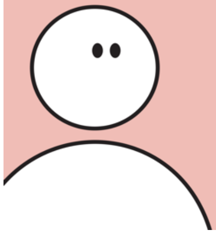

"Doctors know about COPD, but we live with COPD."

"Rehab is really hard to get into, they have long wait lists."

About:

- Actively managing COPD with breathing exercises and health tracking, and uses tools like smartwatch and oximeter regularly to track health.
- Prefers clear explanations and ease of use for health management.
- Appreciates virtual rehab but also believes in-person support is often more effective.

Pain Points ☹

- Struggled to understand the difference between the study app and the virtual pulmonary coaching program app, leading to frustration.
- Was unclear about understanding health data as struggled with what spirometer readings meant or how to use them effectively.
- Virtual pulmonary rehabilitation program was convenient and comfortable but lacked the push of in-person sessions.

Gain Points ☺

- Became more mindful of breathing exercises and their impact.
- Still uses smartwatch and oximeter to track oxygen levels.
- Enjoyed one-on-one coaching for getting answers and support.
- Became more aware of the importance of staying active and exercising regularly.

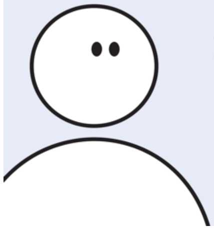

"I would do it again in a heartbeat. It was good listening to other people."

About

- Exercises daily and closely tracks health metrics.
- Tech-savvy, uses a smartwatch and apps, occasionally with help from their spouse.
- Willing to contribute feedback and suggestions for study improvements.
- Prefers direct conversations over digital-only communication.

Pain Points ☹

- Lost access to the study with no notice or follow-up.
- Experienced confusion and lack of support when technical issues occurred.
- Found some exercises too basic and not suited to personal fitness levels.
- No alert system in place to notify researchers of participant struggles.
- The study ended without a structured transition or closure process.

Gain Points ☺

- Continued using the smartwatch for personal health tracking post-study.
- Benefited from virtual pulmonary rehabilitation coaching sessions for motivation and support.
- Gained awareness of the importance of monitoring health data.
- Recognized the need to integrate mental health support in COPD care.
- Interested in contributing to future studies to enhance patient experience.

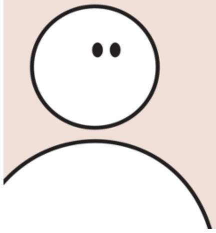

"The study came to a close twice for me... The personal relationship with the counselor and trainer, I got notice. But when my program stopped working, I didn't get any notice on that. So how did I feel about that? Confused, bewildered, stupid, anything you want."

About

- Tech-savvy and interested in health tracking.
- Participated in the study digitally and found smartwatch useful.
- Wants access to personal health data to track progress.

Pain Points ☹

- Unclear instructions on survey frequency and completion process
- Would like more health information or data.
- Participant felt forgotten at times and would have appreciated periodic check-ins.
- Did not fully grasp the specific objectives of the study or how their data was being used.

Gain Points ☺

- Recruitment, onboarding, and offboarding were all described as smooth and non-intrusive.
- Walking more and feeling less out of breath.
- Enjoyed using the smartwatch and considered purchasing one personally.
- Found the study app and smartwatch easy to use.

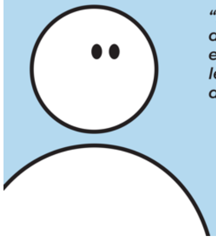

"I've been walking more and getting out, you know, eating right and smoking less. I don't smoke a lot anymore."

About

- Living with COPD for 15+ years.
- Completed in-person rehab
- Interested in virtual pulmonary rehabilitation program but unsure if it will feel the same like the in-person rehab.

Pain Points ☹

- Daily activities can be hard due to changing symptoms.
- Avoids studies that require frequent hospital visits.
- Worries about where health data is shared.
- Prefers short, frequent surveys over long ones.
- Wants more focus on lifestyle changes, not just medication.

Gain Points ☺

- Wants research to focus on real-life challenges like energy and breathlessness.
- Finds tracking symptoms helpful if data is easy to understand.
- Prefers simple, non-intrusive health tools over complicated apps.
- Open to virtual pulmonary rehabilitation program if it's easy to access and structured.

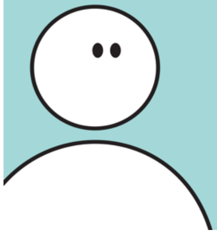

"We're not just lab rats. Keep us informed, involve us in the process."

About

- Relies on home healthcare and strong family support to manage daily needs.
- Prefers digital health programs for medical guidance and connection.
- Stays mentally active with puzzles, diamond art, and other hobbies.
- Finds strength through spirituality and online COPD communities.

Pain Points ☹

- Mobility limitations made even small daily activities challenging.
- Felt disconnected after the study ended.
- Lost access to virtual pulmonary rehabilitation coaching, which had been a valuable source of guidance.
- Anxiety worsened after a 2022 exacerbation.

Gain Points ☺

- Felt a strong sense of safety knowing help was available when needed.
- Gained confidence in tracking personal health data through the study tools.
- Found virtual pulmonary rehabilitation coaching sessions convenient and engaging without the stress of travel.
- Joined online COPD support groups to share experiences and find emotional support.
- Wants to contribute to improving future studies based on personal experience.

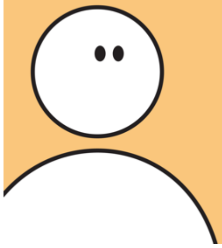

"It saved me at least twice from having to go to the hospital because they could do everything here."

About

- Actively manages COPD using breathing exercises, yoga, and meditation.
- Enjoyed the study and made friends through the virtual pulmonary coaching program.
- Good with technology but likes simple, easy-to-use health apps.

Pain Points ☹

- Felt rushed when the program ended without enough transition time.
- Struggled with smartwatch issues, including false step counts and discomfort from the band.
- Finds medical test results hard to understand and wants clearer explanations in MyChart.

Gain Points ☺

- Learned new ways to manage COPD, like breathing exercises, meditation, and a mucus-clearing device.
- Built a strong support system by starting a self-run COPD group that meets weekly on Zoom.
- Enjoyed using the virtual pulmonary rehabilitation program.
- Discovered new health tools, including a mucus-clearing breathing device.

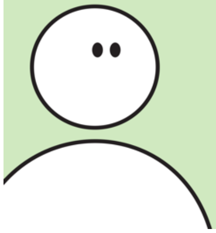

"Medical info needs to be in plain English. Big words don't help if patients can't understand them."

"And you know, it's tough living with a chronic illness, and then when you have other stuff added into it, other health problems, it makes it can make life difficult. But the important part is to always try to focus on the good stuff in life...and hold on to that."

About

- Actively participated in the study and found it beneficial.
- Continues using the smartwatch daily to monitor oxygen levels, sleep, and activity.
- Works part-time and maintains an active lifestyle despite COPD limitations.

Pain Points ☹

- Initially struggled with wearing the smartwatch at night but adapted over time.
- Some COPD-related activities, like climbing hills, were challenging.
- Would have liked more frequent surveys during the study period.
- Feels weather extremes, like cold and humidity, significantly impact COPD but aren't always considered.

Gain Points ☺

- Still uses smartwatch daily to track health and activity.
- Found virtual pulmonary rehabilitation coaching helpful and easy to access.
- Liked checking oxygen levels and health trends.
- Believes stress and mindfulness tracking would be valuable.
- Interested in helping improve future research studies.

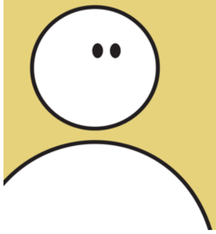

"When it's cold and windy, I have to cover my mouth because it can kind of take your breath away."

"Even when it's really humid out, that's kind of tough too."

About

- Engaged actively in the study and valued the support.
- Stays committed to daily exercise and tracking.
- Manages COPD alongside other health conditions.

Pain Points ☹

- Unsure what some health metrics meant.
- Disappointed not to get final results or progress updates.
- Needed clearer explanations of study findings.
- Medical terms were hard to understand.
- Missed the structure and support after the study.

Gain Points ☺

- Keeps using a smartwatch to track health and stay active.
- Found virtual pulmonary rehabilitation coaching helpful and motivating.
- Learned useful breathing techniques for COPD.
- Wanted clearer feedback on personal health data.
- Open to trying new health-monitoring devices.

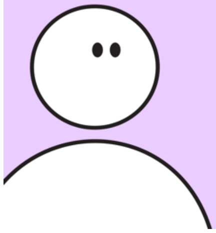

"[MIH] helped me, but I wish I knew what my data really meant."

About

- Retired and participated in the study to stay engaged and wanted to explore ways to manage it better.
- Tech-savvy with an IT background but prefers in-person medical visits over telehealth.
- Values research and is open to participating in future studies.
- Actively uses the MyChart for communication and would prefer study invites that way.

Pain Points ☹

- Felt certain study topics weren't useful for their current health status.
- Found large group sizes in virtual pulmonary rehabilitation sessions made it harder to engage.
- Faced difficulties reactivating and using smartwatch due to account issues.
- Prefers in-person doctor visits for thorough assessments.

Gain Points ☺

- Found the virtual pulmonary rehabilitation sessions helpful for maintaining a fitness routine.
- Started doing more yoga and exercise during the study.
- Appreciated the study's structure as a way to stay active and engaged.

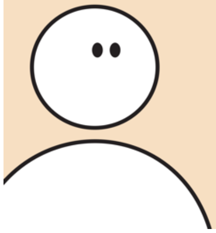

"I didn't expect it, but I exercised less after the study ended."

About

- Takes an active role in managing COPD and tracking health.
- Speaks up for better healthcare and clearer medical information.
- Likes structured health programs but prefers a more personal approach.

Pain Points ☹

- The study felt more like a tech test than a health support program.
- The smartwatch had frequent issues, disconnecting often and causing skin irritation.
- The study app was not user-friendly.
- The study's focus on gadgets did not align with the participant's health needs.
- Health coaching quality varied, with one helpful coach replaced by a less effective one.
- Lack of integration with medical records made it difficult for healthcare providers to access study-related health data.

Gain Points ☺

- Became more aware of breathing and how to manage COPD.
- Found oxygen monitoring helpful for tracking health in real time.
- Enjoyed check-ins and talking about COPD challenges.
- Learned new ways to manage symptoms using biofeedback.

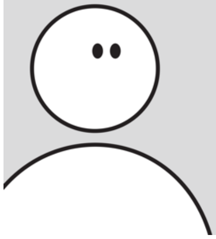

"Managing my healthcare is like a part-time job."
