# Supplementary material for: “A good day is just being able to breathe”: Aligning COPD research with patient needs, a qualitative study
Source: PLoS One. 2025 Sep 16;20(9):e0331403. doi: 10.1371/journal.pone.0331403 (PMC12440184; doi:10.1371/journal.pone.0331403)
Supplement: Attachment — Submitted filename: Response to Reviewer Document.docx [file pone.0331403.s004.docx]

**June 4, 2025**

**Dear Dr. Shaukat,**

**We sincerely appreciate your time and expertise, and that of our reviewers. We are grateful for their thoughtful, critical reviews and believe that responding to their suggestions has significantly strengthened the manuscript.**

**Below, please find a line-by-line response to the reviewers’ comments and the revisions made in response to their suggestions. For clarity, the authors’ responses and actions taken have been bolded. We hope that you find the manuscript substantially improved and suitable for publication.**

1. Please ensure that your manuscript meets PLOS ONE's style requirements, including those for file naming. The PLOS ONE style templates can be found at

<https://journals.plos.org/plosone/s/file?id=wjVg/PLOSOne_formatting_sample_main_body.pdf>

[https://journals.plos.org/plosone/s/file?id=ba62/PLOSOne_formatting_sample_title_authors_affiliations.pdf](https://track.editorialmanager.com/CL0/https:%2F%2Fjournals.plos.org%2Fplosone%2Fs%2Ffile%3Fid=ba62%2FPLOSOne_formatting_sample_title_authors_affiliations.pdf/1/010f01973b519f7d-2d169706-4d96-4f4f-a48c-f94f3aad5cbb-000000/ea7LcZW4q82nTByISOwIKUOYU4msfDxW8yxE-0D4BmM=214)

**Author Response: The authors understand the importance of formatting the manuscript in the style of PLOS ONE.**

**Action Taken: The manuscript has been carefully revised to comply with all PLOS ONE’s formatting requirements.**

2. In the ethics statement in the Methods, you have specified that verbal consent was obtained. Please provide additional details regarding how this consent was documented and witnessed, and state whether this was approved by the IRB

**Author Response: The authors agree that further clarification of our consent process is needed. Because the study is considered less than minimal risk by the authorizing IRB (entailing only activities that would not require written consent outside of the research context), verbal consent was allowed. Per our institutional protocol, a written Fact Sheet was provided to all participants to read and review before deciding to consent and begin study activities. The participant was then allowed to provide verbal consent witnessed by at least one additional study team member, and the interview proceeded. The verbal consent was documented on the cover sheet of each participant’s interview notes. This process was authorized by the supervising IRB of record.**

**Action Taken: The methods section of the manuscript was revised to note that all participants were provided with a written Fact Sheet at the time of recruitment and provided fully informed verbal consent to a member of the study team. A second member of the study team witnessed the consent before the start of the interview. The consent process and participant-facing Fact Sheet were approved under the authorizing IRB and complied with all relevant ethical regulations, including the Declaration of Helsinki.**

3. We noted in your submission details that a portion of your manuscript may have been presented or published elsewhere. [A preliminary version of this manuscript was uploaded to the preprint service MedRXIV.] Please clarify whether this conference proceeding or publication was peer-reviewed and formally published. If this work was previously peer-reviewed and published, in the cover letter please provide the reason that this work does not constitute dual publication and should be included in the current manuscript.

**Author Response: The authors note that the manuscript has been uploaded to a preprint server (MedRVIX), but it has not been formally peer-reviewed, formally published, or presented at an academic conference and therefore this submission does not constitute dual publication.**

**Action Taken: The submission details and cover letter have been updated to note that A preliminary version of this manuscript was submitted to the preprint service MedRVIX, but it has not been formally peer-reviewed, published, or presented at an academic conference.**

4. Thank you for stating the following financial disclosure: [This project was supported by the National Center for Advancing Translational Sciences, National Institutes of Health, through Grant KL2TR00145].

Please state what role the funders took in the study.  If the funders had no role, please state: ""The funders had no role in study design, data collection and analysis, decision to publish, or preparation of the manuscript." If this statement is not correct you must amend it as needed. Please include this amended Role of Funder statement in your cover letter; we will change the online submission form on your behalf.

**Author Response: The authors agree with the editor that it is important to clarify the role of funders in any research project.**

**Action Taken: The cover letter has been revised to note that the project was supported by the National Center for Advancing Translational Sciences, National Institutes of Health, through Grants KL2TR001454 and UL1-TR001453. The funders had no role in study design, data collection and analysis, decision to publish, or preparation of the manuscript.**

5. We notice that your supplementary file are included in the manuscript file. Please remove them and upload them with the file type 'Supporting Information'. Please ensure that each Supporting Information file has a legend listed in the manuscript after the references list.

**Author Response: The authors regret this error**

**Action Taken: The supplemental file has been reuploaded in the appropriate section in the submission portal.**

6. Please review your reference list to ensure that it is complete and correct. If you have cited papers that have been retracted, please include the rationale for doing so in the manuscript text, or remove these references and replace them with relevant current references. Any changes to the reference list should be mentioned in the rebuttal letter that accompanies your revised manuscript. If you need to cite a retracted article, indicate the article’s retracted status in the References list and also include a citation and full reference for the retraction notice.

**Author Response: The citations have been thoroughly reviewed and are correct and complete. In response to one of the reviewers’ feedback, we have added two additional references, which are discussed below.**

**Action Taken: Two additional references were added at the suggestion of a reviewer. No references were removed.**

Reviewers' comments:

**Comments to the Author**

1. Is the manuscript technically sound, and do the data support the conclusions?

The manuscript must describe a technically sound piece of scientific research with data that supports the conclusions. Experiments must have been conducted rigorously, with appropriate controls, replication, and sample sizes. The conclusions must be drawn appropriately based on the data presented.

Reviewer #1: Yes

Reviewer #2: Yes

**Author Response: The authors are glad that the reviewers found the manuscript technically sound.**

**Action Taken: No action taken**

2. Has the statistical analysis been performed appropriately and rigorously?

Reviewer #1: Yes

Reviewer #2: I Don't Know

**Author Response: There was no statistical analysis performed for this qualitative study.**

**Action Taken: No action taken**

3. Have the authors made all data underlying the findings in their manuscript fully available?

The [PLOS Data policy](http://track.editorialmanager.com/CL0/http:%2F%2Fwww.plosone.org%2Fstatic%2Fpolicies.action%23sharing/1/010f01973b519f7d-2d169706-4d96-4f4f-a48c-f94f3aad5cbb-000000/rkm9CIl_2PFU2IxIsj4HGo6bfKeTdpduWNLeSOyGsnc=214) requires authors to make all data underlying the findings described in their manuscript fully available without restriction, with rare exception (please refer to the Data Availability Statement in the manuscript PDF file). The data should be provided as part of the manuscript or its supporting information, or deposited to a public repository. For example, in addition to summary statistics, the data points behind means, medians and variance measures should be available. If there are restrictions on publicly sharing data—e.g. participant privacy or use of data from a third party—those must be specified.

Reviewer #1: Yes

Reviewer #2: Yes

**Author Response: The authors are glad that the reviewers believe that the findings were fully available**

**Action Taken: No action taken**

4. Is the manuscript presented in an intelligible fashion and written in standard English?

PLOS ONE does not copyedit accepted manuscripts, so the language in submitted articles must be clear, correct, and unambiguous. Any typographical or grammatical errors should be corrected at revision, so please note any specific errors here.

Reviewer #1: Yes

Reviewer #2: Yes

**Author Response: The authors are glad that the reviewers feel that the manuscript was written in an intelligible fashion**

**Action Taken: No action taken**

5. Review Comments to the Author

Reviewer #1: Congratulations to the authors on their well-conducted and insightful research.

The authors used the Theoretical Framework of Acceptability (TFA) and the Fogg Behavior Model (FBM) to guide their data collection and analysis, which was apt. The study brings important insights into what matters most to people living with COPD. There are several notable strengths. The methodological rigor is further demonstrated through high inter-coder reliability (Krippendorff’s alpha of 0.86) and evidence of thematic saturation. The qualitative data are rich, and the authors clearly present their findings, supported by explanatory participant quotes.

**Author Response: The authors are glad that the reviewer found the manuscript rigorous and insightful**

**Action Taken: No action taken**

There are, however, a few areas where the manuscript could be improved:

• The FBM mapping is not presented visually. Maybe if the authors could include a figure or some form that illustrates this would help readers better understand how the model was applied.

**Author Response: The authors agree that further explanation of how the FBM model was applied, and how the results were interpreted, would be helpful in the manuscript**

**Action Taken: Additional interpretation of the application of the FBM was added to the discussion. Additionally, we have added “Figure 1”, depicting the mapping of each participant onto an FBM graph to provide readers with an illustration of the results.**

• the manuscript does not provide sufficient detail about who the caregivers are—for example, their relationship to the patient, or the intensity of their caregiving roles. A table or brief summary of this information would be useful.

**Author Response: The authors agree that additional information about the caretakers included in the study would be helpful.**

**Action Taken: A brief summary of the two caretaker participants’ relationship and role with respect to a patient living with COPD was added to the beginning of the results section.**

• The study is based in a single region and includes a sample that appears to be largely White and digitally literate. The limitations section should more clearly acknowledge how this may affect the generalisability of the findings to other populations, particularly those from more diverse or underserved backgrounds..

**Author Response: The authors agree with the reviewer that the relatively homogenous sample represented in this study may limit generalizability to the larger population of patients living with COPD.**

**Action Taken: Additional language was added to the limitations, highlighting that the small, relatively homogenous sample represented in this study may not be generalizable to the entire population of patients living with COPD and that further study specifically evaluating the perspectives of populations underrepresented in research is warranted.**

• It would be helpful if the authors briefly described in the Methods section how the interview guide evolved after piloting.

**Author Response: The authors agree with the reviewer that a description of the interview guide modification process would help readers understand the methodological approach to this study.**

**Action Taken: A description of how and when the interview guide was modified was added to the methods section of the paper.**

• The manuscript mentions participant personas as separate illustrations in the supplementary materials. It would be good to clarify if these were developed solely for internal understanding or were intended for dissemination or further use.

**Author Response: The reviewer asks a good question. The personas developed during the study were for internal use only to spark discussion and guide future patient-centered study design. However, we also felt that they would be of interest to readers and therefore were shared as a supplemental file.**

**Action Taken: The methods were revised to specifically note that the personas were developed to internally guide future study design.**

The authors have done an excellent job of centering patient and caregiver voices in their exploration of COPD clinical research. With a few minor revisions to improve clarity and transparency, I believe this manuscript will make a valuable addition to the literature.

**Author Response: The authors are glad that the reviewer feels that this manuscript will make a meaningful contribution to the literature**

**Action Taken: No action taken**

Reviewer #2: This research explored what matters most to patients with COPD in order to better align COPD research with their real-life needs and experiences. This is a timely and interesting study. I have a few comments:

**Author Response: The authors are gratified that the reviewer found the manuscript relevant and interesting.**

**Action Taken: No action taken**

1. Did you consider the occupation of the participants? Or income? It should have a significant impact on understanding the patient’s needs/perception.

**Author Response: We did not track the income or occupation of the participants. The authors agree with the reviewer that these factors impact patients’ perspectives and that not reporting on them may limit generalizability to the larger population of patients living with COPD.**

**Action Taken: Additional language was added to the limitations, highlighting that the small, relatively homogenous sample represented in this study, as well as the fact that occupation and SES was not reported on, may result in a sample whose perspective is not generalizable to the entire population of patients living with COPD and that further study specifically evaluating the perspectives of populations underrepresented in research is warranted.**

2. Can you please comment on the response rate?

**Author Response: The authors agree with the reviewer that a comment on the response rate would be appropriate in the discussion of this paper. Some participants’ contact information may have been incorrect or out of date, some participants may not be comfortable participating in an interview or using teleconferencing software. We should acknowledge that the response rate**

**Action Taken: The authors added a brief discussion of the modest participant response rate and the possibility of resultant sampling bias to the limitations of the paper.**

3. Prior understanding/research of this topic is not well discussed.

**Author Response: The authors agree with the reviewer that a discussion of prior research exploring this topic was modest in the discussion, and the paper would benefit from additional examination. We are grateful for the suggested citations.**

**Action Taken: The authors added a more robust assessment of the prior literature on the topic of patient priorities in COPD, and compared it to our findings.**

4. In the discussion, it will be impactful to have discussions of possible barriers to addressing the patient’s needs and how to address these issues in real life.

**Author Response: The authors agree that discussing the barriers to patient preferences in more detail would be interesting and important for readers. We did discuss some of the barriers and solutions to real-time data sharing, but agree that we should expand this approach to the study’s other major findings.**

**Action Taken: The authors added exploration of the barriers and solutions to addressing patient preferences in COPD research to the project’s discussion.**

5. Did you consider the subgroup-level analysis? (for example: by gender)

**Author Response: The authors agree with the reviewer that a subgroup analysis of patient perspectives by discrete demographic features would be relevant and interesting. However, as this was a qualitative study, our goal was to explore the depth and range of participant experiences rather than to compare groups. Given the sample size and the exploratory nature of the study, we prioritized thematic saturation and variation across the full dataset rather than stratified comparisons. While subgroup analysis can offer additional insights, our analytic approach focused on identifying shared patterns and meaningful differences across participants to inform patient-centered COPD research design.**

**Action Taken: We included the lack of subgroup analysis as a project limitation**

6. Did you consider key informant interviews? It can guide future implementation of research findings.

**Author Response: Thank you for this thoughtful comment. The focus of this study was to center the lived experiences and preferences of patients with COPD and their lay caregivers in the context of research participation and design. As such, we intentionally limited participants to individuals with direct, personal experience as potential or past research participants, rather than including professional or institutional perspectives. While key informants such as clinicians or researchers may offer valuable implementation insight, they were outside the scope of our study’s aim, which was to elevate patient- and caregiver-driven priorities to inform future COPD research efforts. This is, however, a very good idea for future research.**

**Action Taken: The authors added the involvement of key informants in projects exploring patient-centered research design as a future investigative opportunity.**

Relevant articles:
Sigurgeirsdottir, J., Halldorsdottir, S., Arnardottir, R. H., Gudmundsson, G., & Bjornsson, E. H. (2019). COPD patients' experiences, self-reported needs, and needs-driven strategies to cope with self-management. International journal of chronic obstructive pulmonary disease, 14, 1033–1043. <https://doi.org/10.2147/COPD.S201068>

Poletti, V., Bresciani, G., Banfi, P., & Volpato, E. (2024). Exploring perceptions and expectations of COPD patients: A grounded theory approach for personalized therapeutic interventions. Chronic respiratory disease, 21, 14799731241268262. <https://doi.org/10.1177/14799731241268262>

**Author Response: The authors are grateful to the reviewer for suggesting these highly relevant citations.**

**Action Taken: The suggestions manuscripts were revised, explored, and cited in our revised manuscript.**
